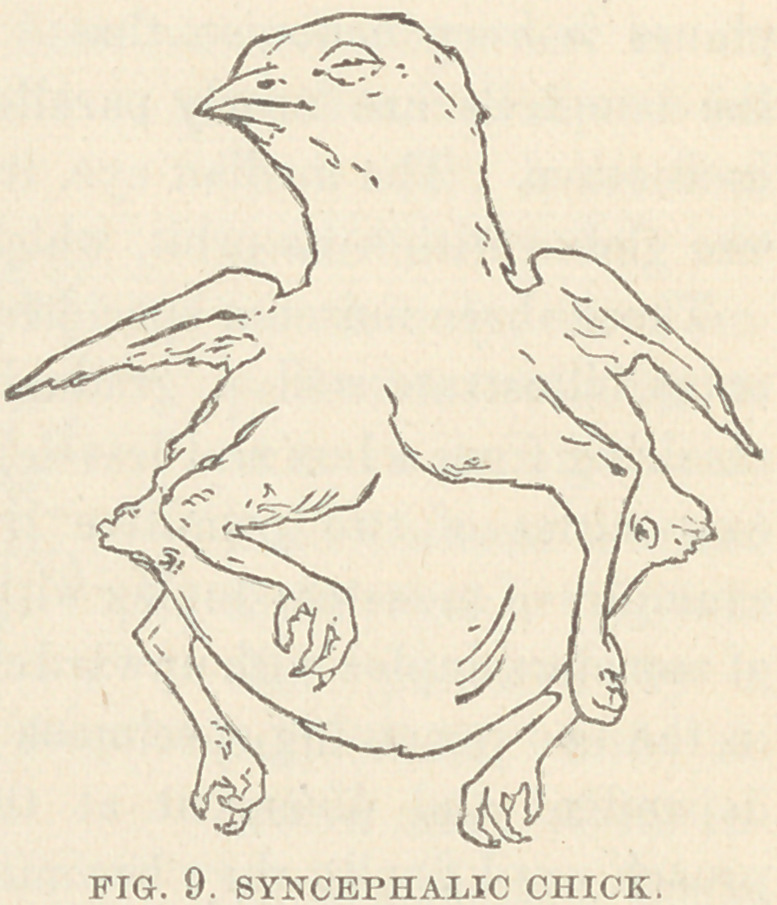# Double Monsters—Description of the Specimens in the Museum of the Brooklyn Anatomical and Surgical Society

**Published:** 1880-04

**Authors:** Lewis S. Pilcher

**Affiliations:** Demonstrator of Anatomy to the Society; adjunct Professor of Anatomy in the Long Island Hospital Medical College


DOUBLE MONSTERS. Description of the Specimens in the Museum of the Brooklyn Anatomical and Surgical Society, with Remarks. By LEWIS S. PILCHER, M.D., Demonstrator of Anatomy to the Society; adjunct Professor of Anatomy in the Long Island Hospital Medical College.

The classification adopted in the present paper is that of Fisher. See Articles on Diploteratology, Transactions of the New York State Medical Society, 1865–66–67 and 68.

This classification is itself based upon the one elaborated by Förster (*Die Missbildungen des Menschen*), whose divisions have been improved and made to follow a more natural order by Fisher.

Double monsters include only beings in which traces of duplicity in the cerebro-spinal axis exist. The method of their development is thus stated by Fisher: "They are invariably the product of a single ovum, with a single vitellus and vitelline membrane, upon which a double cicatricula or two primitive traces are developed. The several forms of double malformation, the degree of duplicity, the character and extent of the fusion, all result from the proximity and relative positions of the neural axes of two more or less complete primitive traces developed on the vitelline membrane of a single ovum."

The clue is thus given to a method of natural classification; the two primitive traces may fuse at their caudal extremity and diverge in varying degrees as they ascend; the result is a double being separated above and joined below, the cleft of the cerebro-spinal axes being from above downward; hence Order I. *Terata katadidyma*, monsters with downward cleavage.

If the fusion is at the cephalic extremity with divergence of the caudal extremities, a double being is produced single above and double below; Order II. *Terata anadidyma*, monsters with upward cleavage.

If the two primitive traces approach at points in their continuity only, while the two extremities diverge, a double being results, double both above and below, with union between; Order III. *Tarata anakatadidyma*, monsters with both upward and downward cleavage.

The differing degrees of duplicity found under each of these orders constitute minor divisions—genera and species—for the nomenclature of which concisely descriptive terms are adopted; the result of this is, that in this classification the name by which any specimen is characterized embodies a fair description of them. I think it merits adoption in preference to any heretofore suggested.

SPECIMEN I. *Order*, Terata katadidyma; *Genus*, diocephalus; *Species*, dibrachius, dipus; *Variety*, diauchenos.

History; mother multiparous; utero-gestation was unattended with anything noteworthy; earlier stages of labor were prolonged; a head was finally delivered by forceps, after which no progress was made for some hours. Dr. Andrew Otterson, having then been called, attempted to introduce his hand into the uterus for exploration; partial version of the retained parts resulted from this attempt, so that the breech engaged; efficient contractions followed, producing expulsion of the breech and body, followed lastly by the second head. The child was dead when delivered. The mother recovered without drawback. Specimen presented by Dr. Andrew Otterson.

FIG. 1.—DICEPHALUS DIBRACHIUS. DR. A. OTTERSON'S CASE.

Description.—External Configuration. A fully developed male child. It has two distinct and perfect heads and necks; one trunk; two upper and two lower extremities.

Its extreme length is 48 centimeters (19 inches.)

The girth of the chest, over nipples, is 39 centimeters ($15\frac{1}{2}$ inches.)

The two heads differ slightly in their size; the circumference of the left head is 36 centimeters ($14\frac{1}{4}$ inches), that of the right head is 33 centimeters ($13\frac{1}{8}$ inches).

There is no anal orifice.

The genitals are male, single and perfect.

Skeleton. The vertebral columns are distinct and perfect throughout; they approach each other gradually from above downward as far as to the lumbar region, whence they run parallel to each other, being separated by a small interval; the sacra, each distinct and perfect, articulate with each other by means of an interarticular fibro-cartilage which unites the contiguous auricular surfaces of the two bones.

From each sacrum springs the innominate bone which forms the wall of the pelvis upon that side; at the symphysis pubis the two unite as usual.

The corresponding dorsal vertebræ of the two columns are united by a series of bony arches formed by coalesced ribs; each arch or compound rib has two normal heads, one at either extremity, by which it articulates with its proper vertebra. The length of these arches decreases from above downwards, the longest being 38 millimeters ($1\frac{1}{2}$ inches) long.

The ribs, which spring from the free sides of the two vertebral columns, are connected to a common single sternum in front by unusually long costal cartilages, and thus complete the thorax.

The clavicle and scapula of either side are normally related to the sternum and ribs.

Resting upon the posterior face of the upper compound ribs, in the middle of the back between the two series of dorsal spines, is a compound scapula formed by the fusion of two bones along their anterior edges; an acromion process, club-shaped, projects forward from the middle of the upper edge of this compound scapula; articulating with this process, and passing directly forward to articulate with the sternum at its upper border, the episternal notch affording an articulating surface, is a slender compound clavicle.

Respiratory system. Two sets of respiratory organs are present, each independent and perfect. There are four pleural sacs. By the blending of the pleural layers which lie in contact in the middle line, a fibro-serous septum is formed which divides the thorax into two cavities posteriorly; these middle pleural sacs and their contents are hidden from view anteriorly by a large pericardial sac, with the posterior wall of which the anterior margin of the septum described becomes blended.

Circulatory System. The pericardium lies in the middle line, directly behind the sternum, and extends to some distance on each side of it. The sac is single, and incloses a compound heart (Fig. 2), the ventricular portions of which remain separate, while the auricles are blended together. Constituting the left mass of this heart are two ventricles and one auricle, the left, which are of normal size, shape and relative position. The origins and relations of the aorta and pulmonary artery upon this side are normal. Into the auricle enter four pulmonary veins. The elements of the right mass are more changed: there is but one ventricle, which however is larger than either of the ventricles of the other mass; from the right side of its base springs a second aorta; there is no pulmonary artery on this side. There is no apparent attempt at differentiation of the auricles; there is simply a single capacious auricle (d), which is blended with the right auricle from the left mass, forming a huge venous reservoir. At the right posterior side of this reservoir enter two small pulmonary veins from the right pair of lungs.

FIG. 2.—COMPOUND HEART.

a. Descending Vena Cava; b. Ascending Vena Cava; c. Right Innominate Vein; d. Compound Auricle.

A single ascending vena cava (b) gathers the blood from the lower portion of the body; above, the left innominate vein of the left child crosses traversely its neck to the point of junction of the two necks, receiving the right internal jugular in its course; here it is joined by the left internal jugular of the right neck, and by a large anomalous vein from behind; the large descending vena cava (a) thus formed descends in a straight course to the middle of the compound auricle. The right innominate vein (c), formed by the right internal jugular and right subclavian veins of the right neck, empties into the compound auricle at its right side.

The two aortas descend each upon the left side of their proper vertebral columns; they do not unite below, nor bifurcate, but each diverging continues as a common iliac, and after giving off the umbilical artery, passes on to be distributed to a lower limb.

Digestive System. There are two stomachs. The left is of normal shape and size, and occupies its usual place in the abdomen. To its cardiac end is attached a normal spleen, the only one present. The right stomach is smaller, pyriform in shape, hidden behind the liver, lies very obliquely, with its pylorus pointing toward the pylorus of the other. Its duodenum joins at once the left duodenum, and the two bowels appear fused together for one sixth of their entire length; a well-marked longitudinal groove so marks the fused bowel that the appearance of a double-barreled gun is produced; transverse section shows that they are divided by a membranous septum into two distinct canals, which communicate freely with each other by frequent openings in the septum; the bowel then divides into two distinct tubes, each with his own mesentery; this persists through a length equal to one third of the whole; then they again fuse, and the double-barrel arrangement persists through a length somewhat greater than at the beginning; the small intestine finally becomes single, and continues thus to its junction with the large intestine, which likewise remains single to its termination; at the

FIG. 3.—STOMACHS AND COMPOUND DUODENUM.

point of beginning of the single tube a small nipple-like diverticulum exists.

The rectum descends to the bottom of the pelvis, where it ends in a cul-de-sac.

The entire length of the small intestine is 1.84 meters (72½ inches); that of the large intestine 66 centimeters (26 inches).

The liver, upon its surface, appears to be a simple organ, but from its posterior inferior border project supernumerary lobes, the evident remains of a second liver. There is but one gall-bladder.

Genito-urinary system. There are three kidneys—a large compound kidney lying in the mid-lumbar sulcus, and one in either lateral lumbar region.

The left kidney is greatly atrophied; the bladder is single; the genital organs single and well developed.

Nervous system. Each head and neck, and each lateral half of the body is supplied by its own cerebro-spinal axis; along the line of fusion only is there any communication between the branches of the two axes.

Remarks. The genus dicephalus, to which this specimen belongs, is characterized by the existence of two distinct and separate heads, either equal or unequal, with various degrees of duplicity in the vertebral column. The component bodies are laterally conjoined; both of the faces look anteriorly, and usually in the same direction. Fisher states that of five hundred cases of human double monsters which are recorded, almost one-third belong to this genus; in these the female sex preponderates in the proportion of about two to one.

Viability. Apart from the accidents of birth, was this monster viable? The answer to this is found in an examination of the structure of the heart and great vessels. Of the compound heart, the left mass displays all the parts of a complete heart, and the arrangement of the great vessels is normal, so that the æration of the blood and its supply to the left child is fully provided for; the right mass is composed of but one auricle and one ventricle; the venous blood passing from the one to the other would have been at once driven on into the right aorta; no pulmonary artery exists to receive even a portion of it for transmission to the right pair of lungs, which, for purposes of aeration, would accordingly have been useless. No inosculation between any large arteries of the two systems exists to have permitted any admixture of arterial blood with the venous current of the right system. This condition would have entailed immediate asphyxia upon the right child, had the monster been delivered alive; the speedy death of the left child would have followed; this specimen was not viable.

Closely related to this specimen, but illustrating a degree of fusion less extensive is the living female double monster known as the St. Benoit twins. In this being the division extends through the thorax as far as to the abdomen; so that the thoracic organs and the upper extremities, as well as the necks and heads,

FIG. 4.—ROSE-MARIE DOURIN, "THE SAINT BENOIT TWINS."

are separate and distinct. During the months of December, 1878, and January and February, 1879, this being was exhibited by its parents in this city. Upon the 28th of February it completed its first year. During this time I visited it repeatedly, but met with much difficulty in any attempt at thorough examination of it from the unreasonable fears and prejudices of the parents. I was finally successful, however, in obtaining an inspection of its whole body, and in enabling Mr. Dickinson to make the sketch which accompanies this report, and which represents well its external configuration.

The names *Marie* and *Rose* have been given to the right and left child respectively. Their patronymic is *Drouin*. In the Canada *Medical and Surgical Journal* of October, 1878, I have since found a description of these beings by Prof. D. C. MacCallum, of Montreal, which, as it corresponds with my own later observation, I repeat:

"Marie is more strongly developed and healthier looking than her sister Rosa, who is smaller, darker and more delicate looking. They are both bright, lively and intelligent looking children. The two bodies, from the heads as far as the abdomen, are well formed, perfectly developed, and in a state of good nutrition. The union between them commences at the lower part of the thorax of each, and from that part downwards they present the appearance of one female child; that is, there is but one abdomen with one navel, a genital fissure with the external organs of generation of the female, and two inferior extremities. The floating ribs are distinct in each, as is also the ensiform cartilage. The lateral halves of the abdomen and the inferior extremities correspond in size and development respectively to the body of the same side; and the same remark applies to the labia majora. The spinal columns are distinct and appear to meet at a pelvis common to both, although the fusion of the children commences at some distance above their junction. From near the extremity of each spine a fissure extends downwards and inwards, meeting its fellow of the opposite side at the cleft between the buttocks near the anus, including a somewhat elevated soft fleshy mass, thicker below than above. At a central point between these fissures, at the distance of two and a half inches [64 mm.] from the point where the vertebral columns meet, and three and a half inches [89 mm.] from the anus, there projects a rudimentary limb with a very movable attachment. This limb, which measures five inches [127 mm.] in length, and is provided with a joint, tapers to a fine point, which is furnished with a distinct nail. It is very sensitive, and contracts strongly when slightly irritated.

"The spinal, respiratory, circulatory and digestive systems of these children are quite distinct. They have each a separate diaphragm, and the abdominal muscles on each side of the mesial line, and the limbs of that side are supplied with blood by the vessels, and are under the control of the nervous system of the corresponding child. They have each a distinct stomach and an alimentary canal, which probably opens at a point close to the common anus. It would follow also that the accessory organs of the digestive system are distinct for each child.

"The two fissures behind are evidently the original clefts between the buttocks of each child, one buttock remaining in its integrity, whilst the other in a rudimentary condition is fused with that of the opposite child, forming the soft fleshy mass from the upper part of which the rudimentary limb projects.

"These children are the products of a second gestation. They were born at St. Benoit, county of Two Mountains, on the 28th February, 1878. The mother is a fine, healthy-looking woman, aged 26 years. Her labor lasted seven hours, commencing at 1 A. M. and terminating at 8 A. M. One head and body were first born; this was shortly followed by the lower extremities, and immediately after the second body and head were expelled."

This being belongs to the genus dicephalus; species tetrabrachius tripus.

The St. Benoit twins have now survived their birth a longer period than in any other recorded instance among the three-footed, four-armed dicephalic monsters. The case recorded by MacLaurin, in the Philosophical Transactions, London, 1723, Vol. XXXII. page 346, which lived for two months, is the next longest lived recorded.

When last examined by myself, this being had attained thirteen months of age; its vital functions were all being performed regularly and properly, and the mental development of the two parts was equal to that usual to children of its age. It apparently had as good an expectation of living to maturity as any other infant.

This being, though strictly included in the species to which I have assigned it, still, in consequence of the very rudimentary character of the third pelvic limb, approaches very closely to the species dicephalus tetrabrachius dipus, which it resembles in its high degree of viability. This rudimentary limb had not grown cor-correspondingly with the rest of the body, and when seen by myself was but little longer than it is described to have been by Dr. MacCallum, ten months before. To this latter species belongs the widely-known case, Ritta-Christina, which died at Paris, November 23d, 1829, having lived eight months and eleven days.

FIG. 5.—RITTA-CHRISTINA.

SPECIMEN II. *Order*, Terata Katadidyma; *Genus*, dicephalus; *Species*, dibrachius; *Variety*, monauchenos.

History: This specimen of two-headed, single-necked monster, with one body and two anterior extremities, is a lamb which was born in the spring of 1874, near Plainfield, N. J. Having been at once discarded by its mother, it was spoon-fed for a time; it received nourishment by one mouth only, some imperfection seeming to exist in the other. It lived between two and three weeks. It was then prepared by a taxidermist, and no record made of its internal structure. It was afterwards secured by Dr. A. R. Matheson, by whom it has been presented to the museum of the society.

FIG. 6.—TWO-HEADED LAMB.

Description : The heads are equal and perfect, as far as external examination shows ; they form an obtuse angle with each other as they spring from the single neck ; the neck externally shows no evidence of duplicity, although undoubtedly there did exist some duplicity in the upper cervical vertebræ. It is to be regretted that the arrangements of the tracheæ and œsophagi cannot be ascertained.

According to Fisher, this an extremely rare variety of duplex formation, so much so that only two or three examples of it have ever been recorded.

SPECIMEN III. *Order*, Terata Katadidyma ; *Genus*, diprosopus ; *Variety*, triophthalmus.

This specimen of double-faced, three-eyed monster, is a small chick, the history of which is unknown. It was presented to the museum by Mr. James E. Pilcher. The accompanying figures admirably exhibit the degree of duplicity present. The angle made by the converging facial planes is very acute, so that the two bills are nearly parallel, and but little separated from each other. The median eye, its palpebæ being single, presents two globes within its orbit, which are fused together.

FIG. 7. DONBLE-FACED CHICK.
*a.* side view. *b* front view of same.

These three museum specimens, together with the Saint Benoit twins, illustrate well a gradually descending scale of duplicity resulting from a less and less degree of divergence of the cephalic extremities of two primitive traces. They are all well-marked examples of monsters duplex with downward cleavage. The order of monsters duplex with upward cleavage is equally well represented in the two remaining specimens in which the vertebral axes, independent and divergent at their caudal ends, gradually approach until finally they become fused at their cephalic ends.

SPECIMENS IV. and V. *Order*, Terata Anadidyma ; *Genus*, syncephalus ; *Species*, monoprosopus.

FIG. 8.—SKELETON OF SYNCEPHALIC DOG.

These two specimems of double-bodied, single-headed, single-faced monsters are identical with each other in their general character. Specimen IV. (Fig. 8.) consists of the skeleton of a duplex puppy which did not survive its birth. It was presented by Dr. J. H. Raymond, and prepared by the writer. The sex was female. There was a common umbilical cord, containing a single umbilical vein; the liver was compound; the stomach and upper two-thirds of the small intestine were single, the intestinal canal then bifurcated and was double to it send; the genito-urinary systems were independent and complete for each body; the thorax contained two perfect sets of lungs; the hearts were fused.

Specimen V. (Fig. 9.) is a duplex chick which was presented by Dr. George W. Baker. Its viscera have not been examined. In these specimens, the head presents no external trace of duplicity; the spinal axes of the compound body, closely juxtaposed at their cephalic extremities, rapidly diverge, and from each of them is developed a complete bony skeleton; these blend so as to form a compound thorax as follows: each lateral half of the sternum with its proper ribs is reflected outward to the right and to the left, and becomes conjoined to the corresponding half sternum of the opposite skeleton. The thoracic cavity thus resulting has a sternum

FIG. 9. SYNCEPHALIC CHICK.

anteriorly, and posteriorly a vertebral column at either side, and walls composed of forty-eight ribs. Four complete anterior extremities spring from this thorax. Each pelvis is separate and distinct, and has its perfectly developed limbs. In the case of the pup the fusion extended as far as to the umbilicus In the case of the chick the fused abdomens form a demi-ovoid body suspended between the four feet of the animal. It did not survive its birth. According to Is. G. Saint Hilaire (1832) this class of monstrosities is comparatively common among animals, but very rare in man, to but two cases of which is he able to refer. Fisher's treatise is still incomplete: the published part of it does not include the syncephali, but in his manuscript, which I have been permitted to examine, I find mention of eight recorded instances of human syncephali.

---

HYDROBROMIC ETHER AS AN ANÆSTHETIC.—The experiments of Rabuteau on the lower animals to demonstrate the anæsthetic properties of hydrobromic ether have, says the *Boston Medical and Surgical Journal*, led Dr. Turnbull of Philadelphia to employ it on the human subject. His successful results have induced others to make use of the substance, and Dr. Lewis has used it for large operations at the Pennsylvania Hospital with gratifying success. Four cases are recorded, viz., amputation of the forearm at the upper third; perineal section for stricture of urethra; resection of stump after former amputation of the arm; and in a case of fracture of both legs, where it was administered in order that a careful examination might be made and tenotomy be performed. The after-effects of the inhalation are less severe than those following the use of chloroform, and the return to consciousness very much more rapid. Though its claims to be admitted into general use are said to be strong, it is admitted that it needs more careful observation to establish it firmly as a safe anæsthetic in general surgery.—*Medical Press and Circular* 14th January, 1880.

## Figures and Tables

**Fig. 1. f1:**
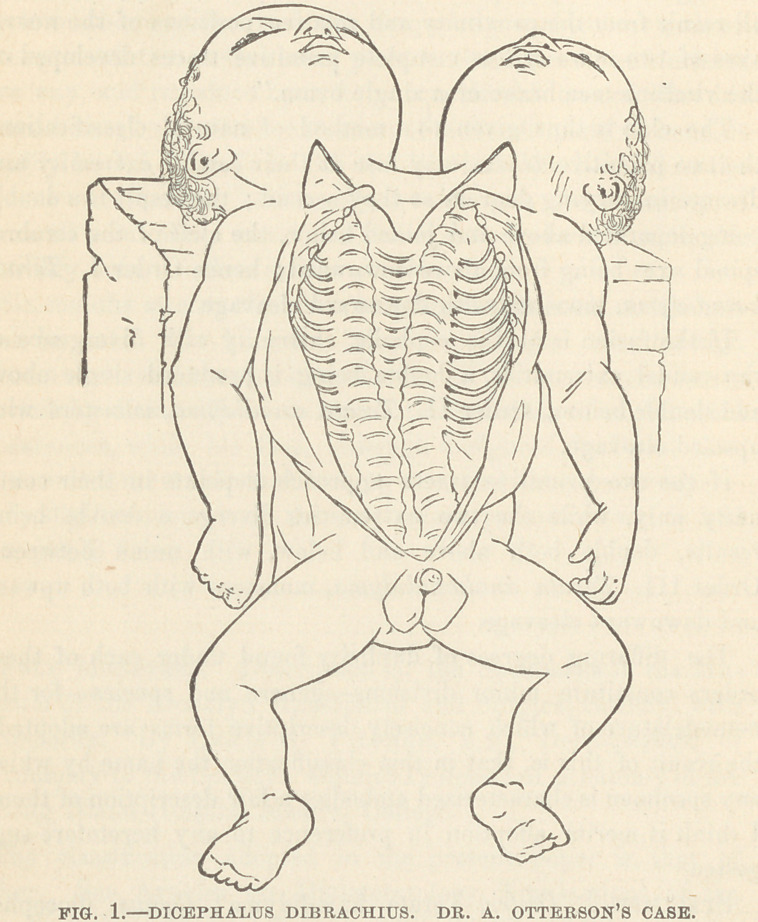


**Fig. 2. f2:**
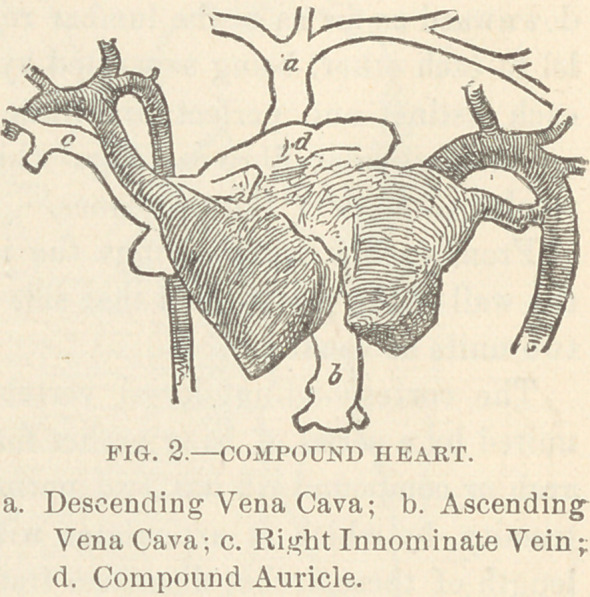


**Fig. 3. f3:**
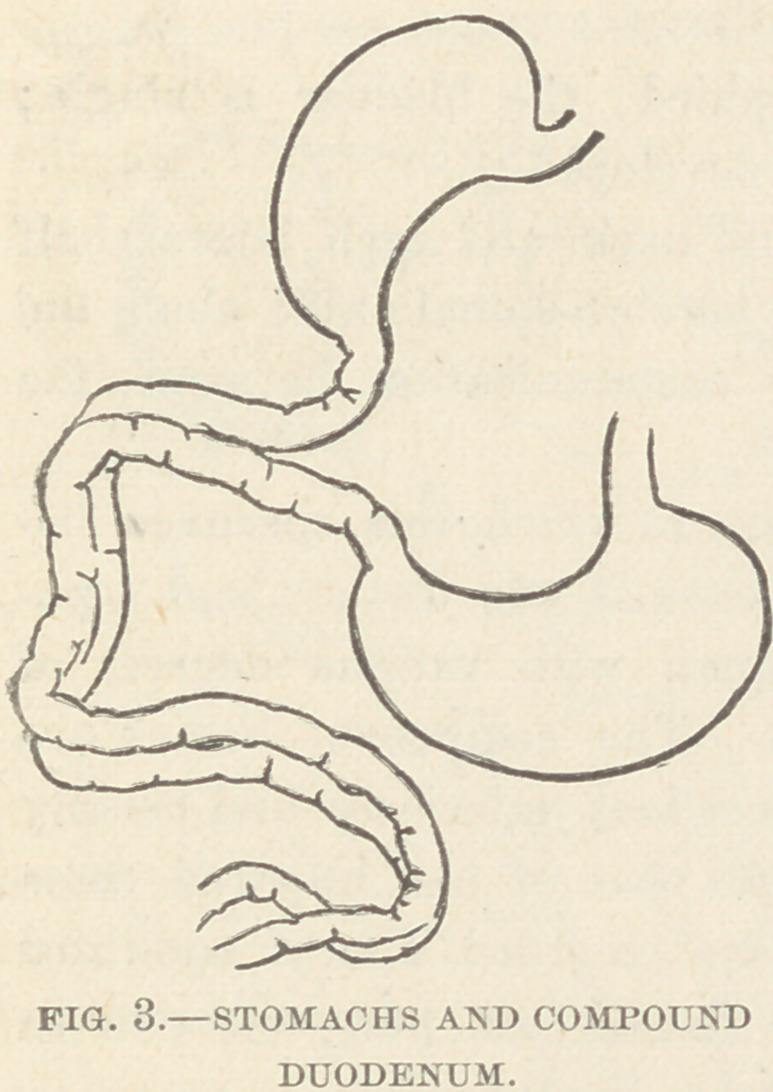


**Fig. 4. f4:**
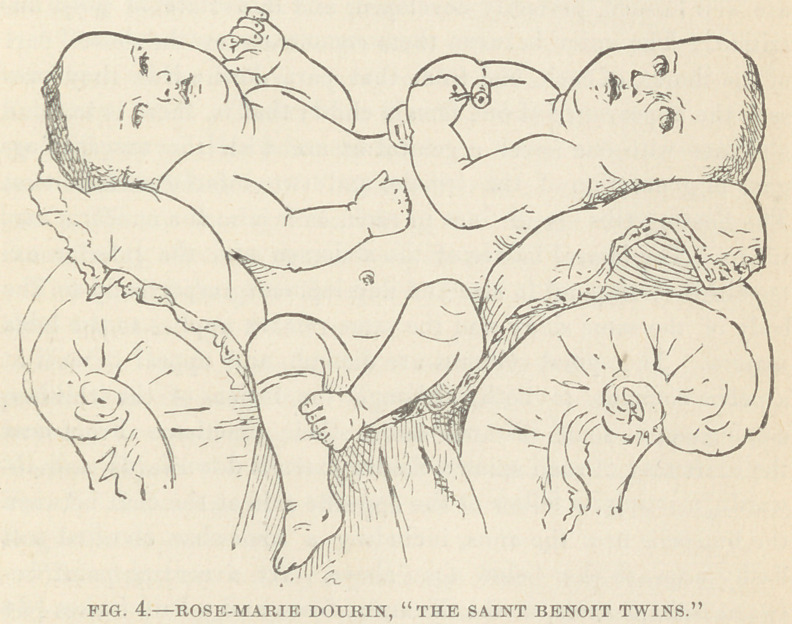


**Fig. 5. f5:**
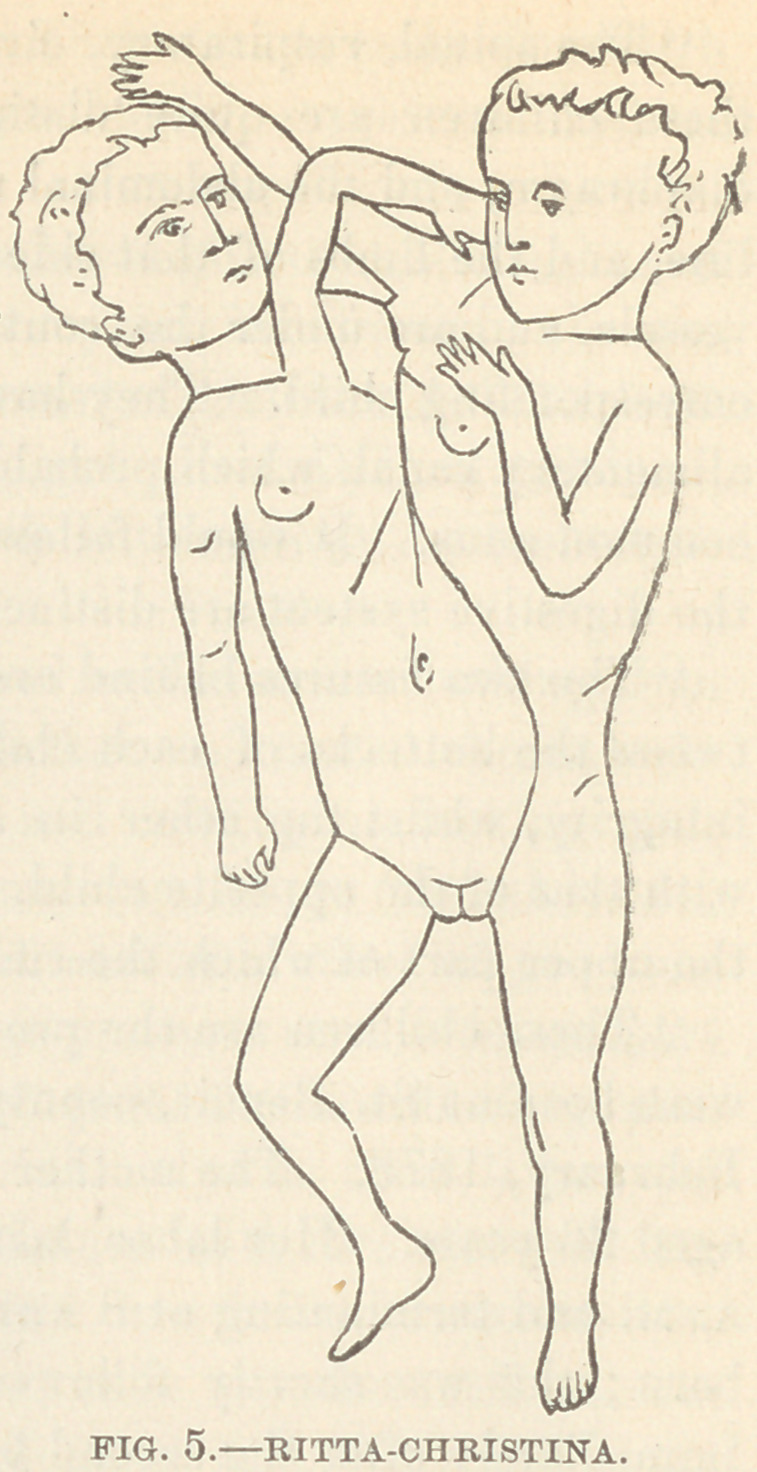


**Fig. 6. f6:**
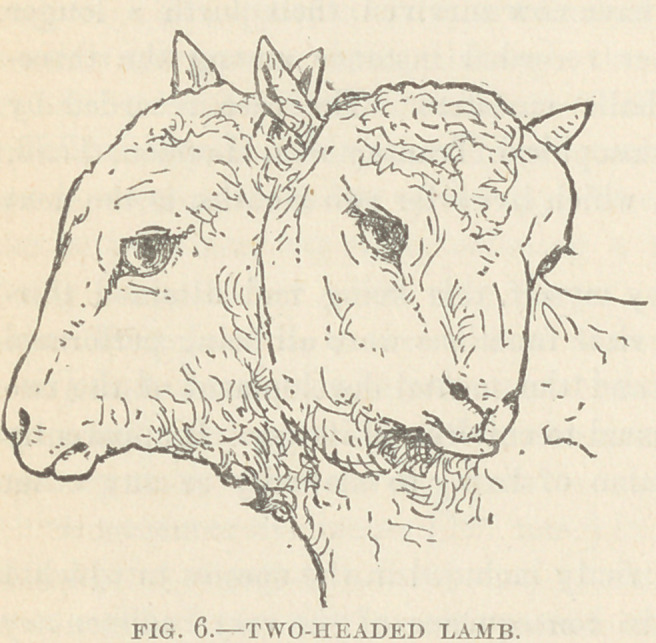


**Fig. 7. f7:**
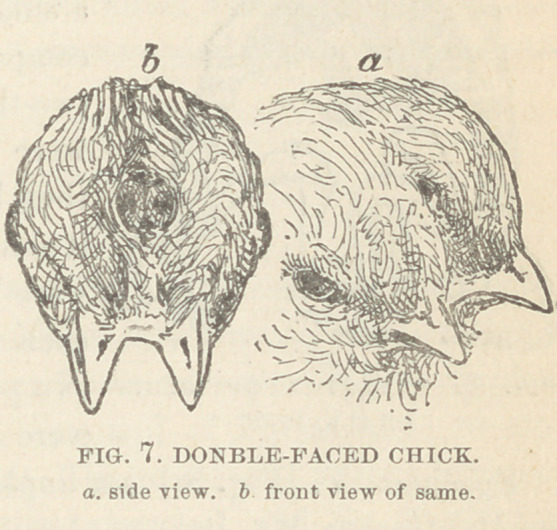


**Fig. 8. f8:**
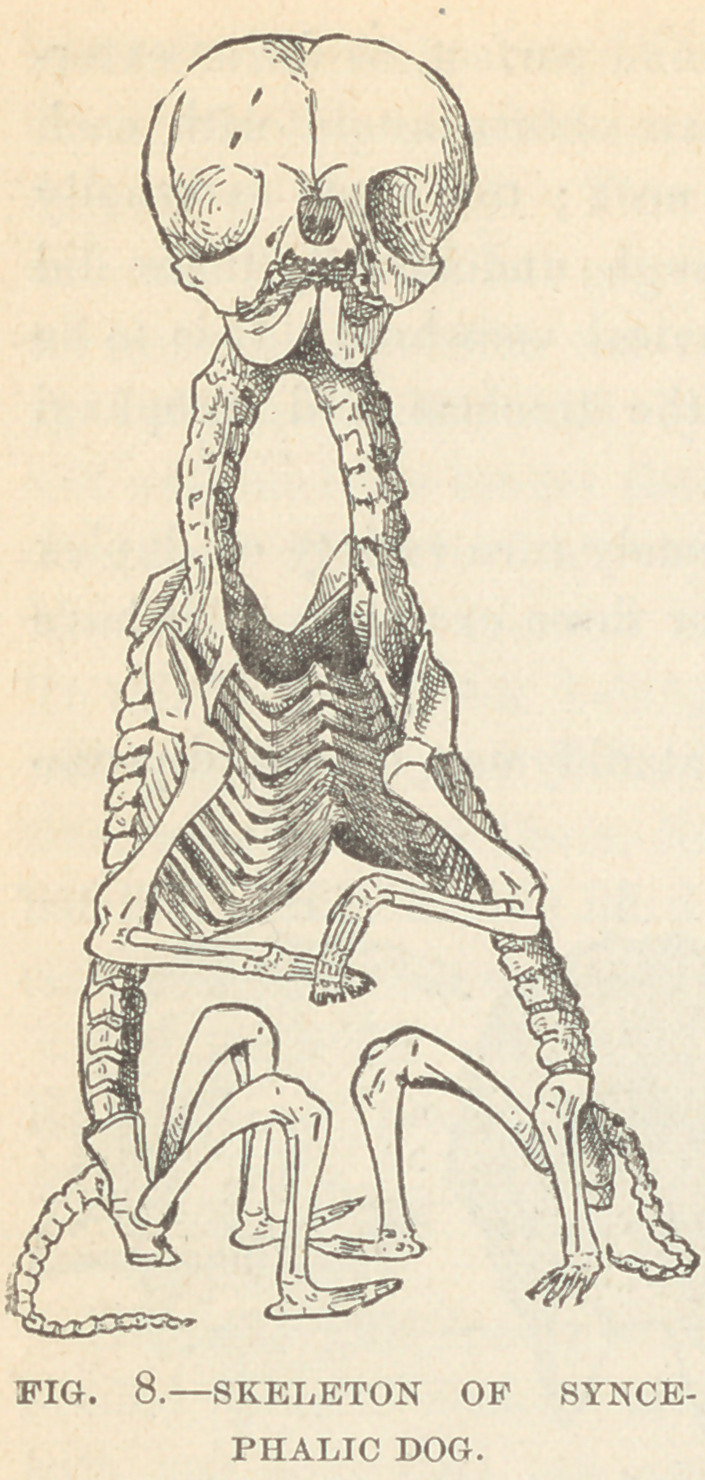


**Fig. 9. f9:**